# Effect of extended visiting hours on physician distractions in the ICU: a before-and-after study

**DOI:** 10.1186/s13054-017-1830-y

**Published:** 2017-09-18

**Authors:** Kay Choong See, Xie Ying Song, Han Tun Aung

**Affiliations:** 1 Division of Respiratory and Critical Care Medicine, University Medicine Cluster, National University Hospital, 1E Kent Ridge Road, NUHS Tower Block Level 10, 119228 Singapore, Singapore; 2 School of Health Sciences, Ngee Ann Polytechnic, 535 Clementi Rd., 599489 Singapore, Singapore

## Main text

Extending visiting hours in adult intensive care units (ICUs) promotes family-centered care, but physicians may be concerned about increased distractions from visitors [1]. We sought empirical evidence within our 20-bed medical ICU, assuming that distractions could cause medical errors [2].

During office hours (07.30 to 17.30 on weekdays; 07.30–12.30 on weekends), two physician teams shared the patient load. Each team comprised one attending physician, one senior resident, and two junior residents. Observations of residents, being front-line medical staff, were performed during two time periods, before and after implementation of extended visiting hours in 2015. For each time period, observations were performed by different groups of six nurse researchers, following a standard method [3]. For each observation session lasting 150–180 min, a pair of observers (A and B) independently recorded the duration, type, source, and severity of distractions. Distractions were defined as breaks in attention, evidenced by observed behaviour such as orienting away from a task or responding verbally [4]. Analysis was based on the data of observer A only, while reliability was assessed using the data from observer B. All physicians gave informed consent to be observed, and no one declined participation. Ethics approval was obtained (DSRB/2011/00279).

From 11 May to 26 June 2011 (previously reported [3]), visiting hours were restricted to 12.00–14.00 and 17.00 to 20.00 (total 5 h), and from 8 May to 9 July 2017, visiting hours were extended to 09.00–21.00 (total 12 h). Mean distraction frequency did not differ between both time periods (4.36 ± 2.27/h versus 5.00 ± 2.68/h, *t* test *P* = 0.262), even after adjusting for resident seniority using multiple linear regression (*P* = 0.303). The distribution of current activities and distraction characteristics differed, though predominant type, sources, and severity of distractions were similar (Table 1). The duration of distractions was short, and median duration per distraction was shorter in the later time period (2 min versus 1 min, *P* < 0.005). Reliability, as assessed by agreement of all observed distractions between observers A and B, was excellent in both time periods (99.1% and 96.1%, respectively).

**Table 1:** Characteristics of distractions

Variables studied	Restricted visiting hours	Extended visiting hours	*P* value
Sessions observed	38	39	NA
Total observation time, h	100.4	117	NA
Number of distractions	444	585	NA
Start time of sessions observed			
Morning (07.30–12.00), *n* (%)	23 (60.5)	21 (53.8)	0.554
Afternoon (12.00–17.30), *n* (%)	15 (39.5)	18 (46.2)	
Frequency of distractions/h, mean ± SD	4.36 ± 2.27	5.00 ± 2.68	0.262
Distraction duration (min), median (IQR)	2 (2–4)	1 (1–2)	< 0.001
Current activity at the time of distraction, *n* (%)			< 0.001
Writing notes	97 (21.8)	150 (25.6)	
Conducting ward round	84 (18.9)	35 (6.0)	
Entering treatment orders	75 (16.9)	148 (25.3)	
Reading notes	61 (13.7)	162 (27.7)	
Talking to a colleague	47 (10.6)	49 (8.4)	
Examining a patient	37 (8.3)	11 (1.9)	
Entering medication orders	14 (3.2)	3 (0.5)	
Performing non-sterile procedure	11 (2.5)	7 (1.2)	
Performing sterile procedure	9 (2.0)	9 (1.5)	
Talking to a patient	3 (0.7)	4 (0.7)	
Talking to a patient’s relative	3 (0.7)	6 (1.0)	
Performing resuscitation	2 (0.5)	0 (0.0)	
Giving medications	1 (0.2)	1 (0.2)	
Type of distraction, *n* (%)			<0.001
Asked to speak to colleague	177 (39.9)	367 (62.7)	
Asked to write treatment orders	61 (13.7)	43 (7.4)	
Asked to attend to a patient	61 (13.7)	25 (4.3)	
Asked to sign a document	31 (7.0)	5 (0.9)	
Going to the toilet/going elsewhere	30 (6.8)	89 (15.2)	
Asked to perform a procedure	29 (6.5)	7 (1.2)	
Asked to speak to a patient’s relative	25 (5.6)	18 (3.1)	
Drinking/eating	21 (4.7)	14 (2.4)	
Asked to write medication orders	7 (1.6)	13 (2.2)	
Asked to administer medications	2 (0.5)	4 (0.7)	
Source of distraction, *n* (%)			0.026
Other doctor	156 (35.1)	207 (35.4)	
Nurse	135 (30.4)	147 (25.1)	
Self	83 (18.7)	164 (28.0)	
Phone call	30 (6.8)	28 (4.8)	
Other healthcare worker	24 (5.4)	21 (3.6)	
Relative	14 (3.2)	15 (2.6)	
Patient	1 (0.2)	2 (0.3)	
Monitor alarm	1 (0.2)	1 (0.2)	
Severity of distraction, *n* (%)			<0.001
No effect on activity	13 (2.9)	82 (14.0)	
Momentary pause^a^	136 (30.6)	193 (33.0)	
Complete pause^b^	210 (47.3)	288 (49.2)	
Abandons activity, attends to distraction	85 (19.1)	22 (3.8)	

^a^Activity resumes during distraction

^b^Activity resumes only after distraction ceases

*IQR* interquartile range, *NA* not applicable, *SD* standard deviation

Overall, distractions among ICU doctors were common (~4–5 distractions/doctor/h), and this is consistent with data from other studies using different observation methods [5]. There was also no significant increase in the frequency of distractions after implementation of extended visiting hours in the ICU. Being asked to speak to family members constituted a small proportion (<5%) of the distractions, and therefore our study did not provide empirical support for the concern of increased distractions from visitors due to extended visiting hours.

## Abbreviation

ICU: Intensive care unit
